# Super-resolution imaging to reveal the nanostructure of tripartite synapses

**DOI:** 10.1042/NS20210003

**Published:** 2021-10-14

**Authors:** Natalija Aleksejenko, Janosch P. Heller

**Affiliations:** 1 School of Biotechnology and National Institute for Cellular Biotechnology (NICB), Dublin City University, Glasnevin, Ireland; 2 Queen Square Institute of Neurology, University College London, London, United Kingdom

**Keywords:** astrocytes, super-resolution microscopy, synapses, tripartite synapses

## Abstract

Even though neurons are the main drivers of information processing in the brain and spinal cord, other cell types are important to mediate adequate flow of information. These include electrically passive glial cells such as microglia and astrocytes, which recently emerged as active partners facilitating proper signal transduction. In disease, these cells undergo pathophysiological changes that propel disease progression and change synaptic connections and signal transmission. In the healthy brain, astrocytic processes contact pre- and postsynaptic structures. These processes can be nanoscopic, and therefore only electron microscopy has been able to reveal their structure and morphology. However, electron microscopy is not suitable in revealing dynamic changes, and it is labour- and time-intensive. The dawn of super-resolution microscopy, techniques that ‘break’ the diffraction limit of conventional light microscopy, over the last decades has enabled researchers to reveal the nanoscopic synaptic environment. In this review, we highlight and discuss recent advances in our understanding of the nano-world of the so-called tripartite synapses, the relationship between pre- and postsynapse as well as astrocytic processes. Overall, novel super-resolution microscopy methods are needed to fully illuminate the intimate relationship between glia and neuronal cells that underlies signal transduction in the brain and that might be affected in diseases such as Alzheimer’s disease and epilepsy.

## Introduction

Information processing in the central nervous system occurs mainly at synapses, the connection points between neurons. An intimate relationship between neurons and glial cells underlies proper synaptic function. Astroglia in particular play essential roles in information processing. These cells are not only needed for structural support but astroglia also fulfil several other indispensable functions. These include clean-up of brain debris, digestion of dead neurons and pruning of synapses [1] as well as being the drivers of the glymphatic system, the major waste clearing system in the brain [2–4]. Moreover, related to synaptic function, astrocytes express many powerful neurotransmitter and ion channels as well as transporters. These allow them to sustain homeostasis in the brain through processes such as glutamate uptake [5,6] and potassium buffering [7,8]. Moreover, astrocytes release gliotransmitters that directly impact synaptic function [9,10]. These processes are dysregulated in many disorders, including Alzheimer’s disease and epilepsy [11,12].

In the brain, astrocytes adopt a sponge-like morphology, featuring a few stem processes and thousands of thin protrusions that emanate from these stem processes [13,14]. While these fine protrusions permeate the extracellular space, there is minimal overlap between territories formed by individual cells [15,16]. This is surprising as astrocytes are interconnected through gap junctions, forming a syncytium that tiles the brain [15,16]. Although astrocytes are electrically passive, they signal through Ca^2+^ waves and through the aforementioned syncytium [17–19]. Through this calcium signalling, the cells are able to integrate and transmit physiological signals among neuronal and glial cells [14,20,21].

The fine protrusions can either form astrocytic endfeet and encircle endothelial cells as part of the neurovascular unit of the blood–brain barrier or they can enwrap excitatory synapses throughout the brain. These processes are often called perisynaptic astrocytic processes (PAPs) and form part of the so called tripartite synapse, together with pre- and postsynaptic structures [22–25]. Depending on the brain region and the physiological state of the animal this synapse coverage varies [for reviews see 13,26]. There is a constant molecular exchange between glial cells and neurons, shaping synaptic transmission and regulating use-dependent plasticity, in the healthy brain as well as in pathological states [9,20,26–29].

Due to the nanoscopic nature (their diameter can often be smaller than the diffraction limit of light) of the PAPs, researchers have had to rely on electron microscopy (EM) to visualise and investigate tripartite synapses [14,30,31]. EM revealed the intricate relationship between neuronal, synaptic structures and astrocytes processes (e.g. reviewed in [13]). It was also shown using EM that PAPs preferentially approach thin spines over larger spines [32].

PAP coverage of synapses depends on several factors and differs depending on the physiological state, local neuronal activity, synaptic plasticity, or certain behaviours [33–39].

Confocal microscopy, which is diffraction limited, has been used to explore the dynamic nature of PAPs in real time [40–43]. The dynamic nature of PAPs has also been shown with super-resolution microscopy approaches (see below).

In this review, we will provide an overview and discussion of recent developments in tripartite synapse imaging using super-resolution methods. We will also provide a short introduction into different super-resolution methods and recent advances in revealing the nanostructure of neuronal synapses. However, for further information we point the reader to some excellent, recent reviews on super-resolution microscopy and also its use in neuroscience research [44–53].

## Super-resolution methods

As mentioned above, confocal microscopy (as well as other types of light microscopy) is diffraction limited, meaning that discerning individual molecules can be difficult. EM, which has traditionally been used in order to image nanoscopic molecules, comes with several disadvantages, including its exclusive use for fixed tissue specimens. In addition, it is labour and time intensive, and correlational comparisons between different samples are difficult to attain and interpret. This is also true for some of the super-resolution methods described below.

Super resolution imaging allows the user to image beyond the diffraction limit of light while using traditional sample immunostaining methods associated with fluorescent light microscopy. Several super resolution methods have been developed, each with its own advantages and disadvantages. However, the discussion of these is beyond the scope of this review and the authors again point to some excellent reviews of this topic [44–53]. Most widely used methods include stimulated-emission depletion (STED) microscopy [54], structured illumination microscopy (SIM) [55], single molecule localisation microscopy (SMLM) [56–58] and expansion microscopy (ExM) [59,60]. Most of the super-resolution imaging is usually performed on fixed specimens. However, STED and SIM are suited for live cell and live tissue imaging (see below). Moreover, some SMLM adaptations such as single particle tracking PALM (sptPALM) and universal point accumulation for imaging in nanoscale topography (uPAINT) can be used to track single-molecule trajectories at very high densities, for short periods of time [46].

STED microscopy relies on a second, doughnut-shaped excitation beam which depletes excitation at the periphery of the primary excitation beam, hence narrowing the emission spot and allowing resolution beyond the diffraction limit [54]. One of the advantages of using STED is that there is no need for further computational processing, as is the case in other super-resolution microscopy methods. This reduces the risk of artifact generation. However, as this method requires high laser intensity, it runs the risk of bleaching the sample as well as causing physical damage to live cells [49,50,53].

SIM relies on bar code-like patterns which are shifted and rotated in the excitation path [55]. This creates high-frequency information which can be used to reconstruct a super-resolved image. As mentioned, SIM is particularly well suited for imagine live samples. However, as multiple images need to be taken at high intensities, this method runs the risk of damaging live samples, similar to STED. SIM can be expensive to set up, as it requires a dedicated microscope [53].

SMLM encompasses several different techniques that all rely on a similar molecular mechanism - pinpointing the point source of fluorescence (which can be one fluorophore/molecule) in each imaging cycle through repeated stochastic excitation of only a small, sparsely distributed subset of fluorescent molecules [58]. After acquiring several thousand frames a super-resolved image can be reconstructed. SMLM includes photo-activated localisation microscopy (PALM) and stochastic optical reconstruction microscopy (STORM) [56,57]. These images can be taken with a regular fluorescent microscope and there are many free software options available in order to process these images, making it one of the more easily accessible super-resolution microscopy methods [50]. However, due to the processing of these images, it can be prone to artifact generation [61].

ExM uses literal expansion of the tissue by a factor great enough to separate individual molecules of interest beyond the diffraction limit, followed by imaging with conventional microscopes such as confocal microscopy [59,60]. This method is relatively inexpensive and easy to implement. However, it is only suitable to be used on fixed samples [62].

## Super-resolution imaging of neuronal synaptic structures

Several advances have been made in our understanding of neuronal synapses using super-resolution microscopy (for recent reviews see [45–48,52]).

Especially STED microscopy has been employed to analyse the composition of presynaptic structures and vesicle dynamics within, both *in vivo* and *in vitro* [63–67], showing for example that about 75 densely packed plasma membrane soluble N-ethylmaleimide-sensitive factor attachment protein receptor (SNARE) proteins form clusters of ∼60 nm diameter in PC12 cells [68]. SMLM was later used to investigate the nanoclustering behaviour of individual molecular players at the presynapse *in vivo* and *in vitro* [69–72].

STED as well as SMLM have been used to image dendritic spines and postsynaptic structures in cultured cells and brain slices [73–79]. Recently, STED has been employed to illuminate spine dynamics in living mice [80–86]. These developments now make it possible to image up to three labels in the cortex of living mice [85], and to chronically illuminate the same super-resolved structures over a time course of up to one month [86]. Besides revealing more macroscopic structures of spines and the underlying cytoskeletal structures, super-resolution microscopy has been used to further our understanding of the nanoscale organisation of the postsynaptic density (PSD).

Seminal work published in 2010 provided a detailed three-dimensional map of the nanoscale structure of excitatory synapses in fixed murine brain sections using SMLM [87]. The group also compared activity-dependent changes in the organisation of N-methyl-D-aspartate receptors (NMDARs) and α-amino-3-hydroxy-5-methyl-4-isoxazolepropionic acid receptors (AMPARs) across the mouse olfactory bulb [87].

Using different super-resolution microscopy techniques, it was shown that AMPARs are packed in nanoscopic clusters in cultured cells [88–90]. These clusters are smaller than the PSD (0-4 AMPAR nanodomains per PSD, with an average size of ∼80 nm, and 20–25 AMPARs per nanodomain), and their number depends on the size of the PSD. The researchers also found that PSD95 molecules show clustering behaviour in nanodomains of ∼150 nm diameter within each PSD in mouse brain tissue and in cultured cells [88–92].

Likewise, the nanoscale clustering of the scaffolding protein gephyrin as well as of glycine and gamma-aminobutyric acid (GABA) type A receptors at inhibitory synapses has been investigated [93–95]. Using quantitative 3D-PALM, Specht and colleagues measured that 40–500 gephyrin molecules are packed at a density of about 5000 molecules/µm^2^ in cultured spinal cord neurons, and that *in situ* about three times as many gephyrin molecules are packed more densely [94].

Recent beautiful work combined super-resolution microscopy imaging with plasticity induction and revealed a trans-synaptic organisation of scaffolding molecules both at excitatory and at inhibitory synapses in organised tissue and in cultured cells [92,93,95–97]. These groups found that presynaptic proteins such as RIM1/2 form nanodomains in similar ways to postsynaptic scaffolding proteins PSD95 and gephyrin. These nanodomains are aligned in nanocolumns across the synaptic cleft, which remain intact after plasticity induction.

## Super-resolution imaging of tripartite synapses

As discussed above, astrocyte processes and especially PAPs display nanoscopic features. Early super-resolution studies did not focus on the relationship between synapses and astrocytic molecules but rather on the subcellular localisation and clustering behaviour of individual proteins of interest. For example, Verkman and colleagues investigated the clustering behaviour of aquaporin 4 and of the inwardly rectifying potassium channel Kir4.1 using SMLM techniques in cultured cells and in brain sections [98–100]. Moreover, thin astroglial processes have been illuminated by expressing genetically-encoded fluorescent proteins and by labelling glutamine synthetase and S100β in cultured cells and brain sections [34,101–105].

**Figure 1:**
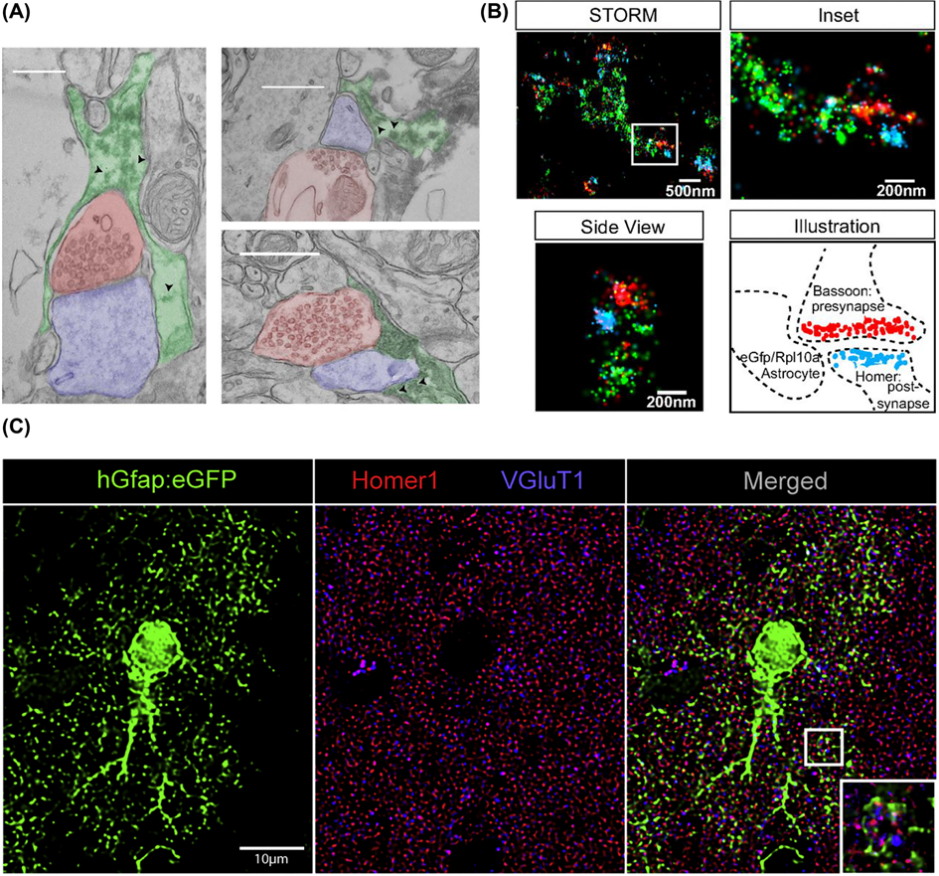
EM, SMLM and STED images visualising astrocyte processes in the vicinity of excitatory synapses (**A**) EM image of diaminobenzidine-labelled EGFP/RPL10A (arrowheads) in astrocyte processes (green) near cortical synapses (axon = red and PSD = blue); scale bars = 500 nm; modified from [106] with permission. (**B**) SMLM image showing an EGFP/RPL10A (green) filled astrocyte process near a synapse (presynaptic bassoon (red) and postsynaptic Homer (blue); inset of box on left, and side view is a 90° rotation of a second synapse; modified from [106] with permission. (**C**) Deconvoluted confocal microscopy image of an astrocyte (hGfap-eGFP, green), presynaptic vGluT1 (blue) and postsynaptic Homer1 (red); the magnified area shows the STED image for vGluT1 and Homer1 merged with deconvoluted confocal image for eGFP; scale bar = 10 µm; modified from [107] with permission.

Recent studies have investigated translation events that occur locally in astrocytic processes in the vicinity of synapses [106–108]. EM and SMLM have been used to reveal the presence of Rpl10a, a component of the 60S ribosomal subunit, in close apposition to synapses in mouse brain sections [106] (Figure 1A,B). Local translation in astrocytic processes has been implicated in fear conditioning [107], and its impairment might contribute to the development of amyotrophic lateral sclerosis [109]. Here, researchers used STED and confocal microscopy to visualise astrocyte processes in the vicinity of excitatory synapses in mouse brain slices [107] (Figure 1C). The presence of mRNA and local translation has also been shown to occur in astrocytic endfeet surrounding blood vessels in the brain [110].

Using STED and SIM, researchers have shown that neuronal activity increased connexin 30 expression and localisation in perisynaptic processes in hippocampal astrocytes in mouse brain slices [111]. The same group recently showed that astrocytes close the critical period for visual plasticity via developmental upregulation of connexin 30 which in turn inhibits expression of the extracellular matrix degrading enzyme matrix metalloproteinase 9 [112].

In a recent beautiful publication, researchers have used a plethora of techniques including an *in vivo* chemico-genetic approach that applies a cell-surface fragment complementation strategy, Split-TurboID and STED discovering that neuronal cell adhesion molecule (NRCAM) is expressed in cortical astrocytes where it localises to PAPs and restricts their neuropil infiltration [113]. Astrocytic NRCAM interacts with neuronal NRCAM coupled to gephyrin at inhibitory postsynapses, and its deletion reduces the number of inhibitory synapses and decreases inhibitory synaptic function with minimal effects on glutamatergic synaptic density or overall excitation in mice [113].

Recently, researchers visualised bestrophin-1 (Best1) expression at tripartite synapses using lattice SIM and found that, in brain tissue from wild-type mice, astrocytic Best1 localises closer to glutamatergic synapses than to GABAergic synapses [114]. In APP/PS1 mice (Alzheimer’s disease model) tissue, however, Best1 resides further away from glutamatergic synapses and closer to GABAergic synapses [114].

In a recent preprint, Südhof and colleagues used SMLM in mouse brain sections to decipher the role of astrocytic neurexin-1 in synapse function and maturation [115]. The authors showed that neurons as well as astrocytes express neurexin-1, which is organised in discrete nanoclusters at excitatory synapses. Distinct heparan sulphate modifications and alternative splicing lead to different ligand specificities and enables compartment-specific neurexin-1 signalling. Even though deletion of neurexin-1 from either astrocytes or neurons did not have an effect on synapse numbers, the authors found that neuronal neurexin-1 is required for NMDAR-mediated synaptic responses, and that astrocytic neurexin-1 is needed for silent synapse maturation, AMPAR recruitment and long-term potentiation [115].

Recent work utilising STED imaging showed that in spinal cord sections 56% of synapses (PSD95 and vGluT2 or vGluT1 pairs) are association with GLT-1 (the major astrocytic glutamate transporter), 30% with glial fibrillary acidic protein and 14% with phosphorylated ezrin [116]. PSDs associated with an astrocytic protein are larger in size and fluorescent intensity. Furthermore, PSDs that comprised more than one PSD95 nanocluster are more likely to be part of a tripartite synapse, and tripartite nanoclusters appear brighter and hence more enriched in PSD95 [116].

**Figure 2:**
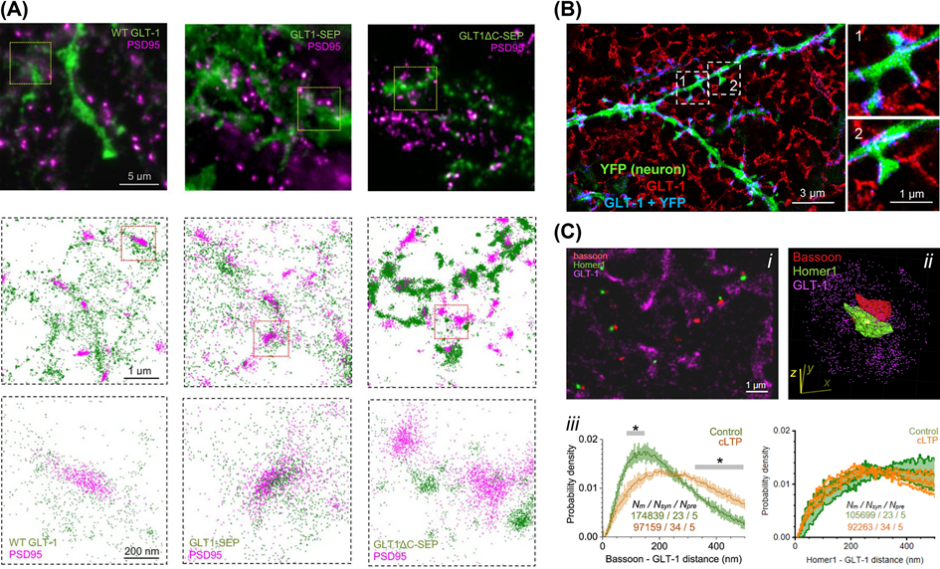
SMLM and ExM images highlighting GLT-1 distribution around synapses (**A**) Wide-field fluorescent images highlighting GLT-1 (green) and PSD95 (magenta) in mixed hippocampal astroglia-neuron cultures (top row). SMLM images showing individual labelled GLT-1 (green) and PSD95 (magenta) localisations (middle row depicting yellow squares in top row; bottom row depicting red squares in the middle row); modified from [117] with permission. (**B**) ExM image of dendrite and spines of a CA1 pyramidal neuron (green, YFP) and surrounding GLT-1 (red) labelling; with regions of ‘colocalisation’ highlighted in blue; modified from [119] with permission. (**C**) SMLM images highlighting localisations of presynaptic bassoon (red), postsynaptic Homer 1 (green) and astrocytic GLT-1 (magenta) (i and ii). Nearest-neighbour distances (probability density, mean ± SEM) between GLT-1 and bassoon or Homer1 (in control tissue (green) and ∼30 min after chemical long term potentiation (cLTP) induction (brown)) (iii); sample size: *N*_m_, inter-molecular distances; *N*_syn_, synapses; *N*_pre_, slices; SEM relates to *N*_pre_ = 5; ^∗^*P*<0.05 (grey segments, significant difference), modified from [34] with permission.

In a recent publication, we used fluorescence recovery after photobleaching in organotypic rat hippocampal sections to demonstrate that 70–75% of GLT-1 molecules dwell on the astrocyte surface, recycling with a lifetime of ∼22 s [117]. Using SMLM in cultured rat hippocampal cultures, we showed that GLT-1 surface expression and clustering behaviour relies on its C-terminus and its deletion accelerates GLT1 membrane turnover (Figure 2A) [117].

Recent attempts to study astrocyte processes and glutamate uptake have utilised ExM of mouse brain sections [118,119]. The authors showed that larger spines had reduced glutamate uptake efficiency which correlated with greater GLT-1 levels (Figure 2B) [119].

To further analyse the distribution of glutamate transporters surrounding excitatory synapses, we used a 3D SMLM protocol of brain sections [34,120,121]. Through a series of experiments involving optical glutamate sensors, patch clamp electrophysiology, sensory stimulation and super-resolution microscopy, we showed that induction of long-term potentiation leads to withdrawal of PAPs from excitatory synapses which in turn boosts glutamate escape and inter-synaptic cross-talk (Figure 2C) [34].

**Figure 3:**
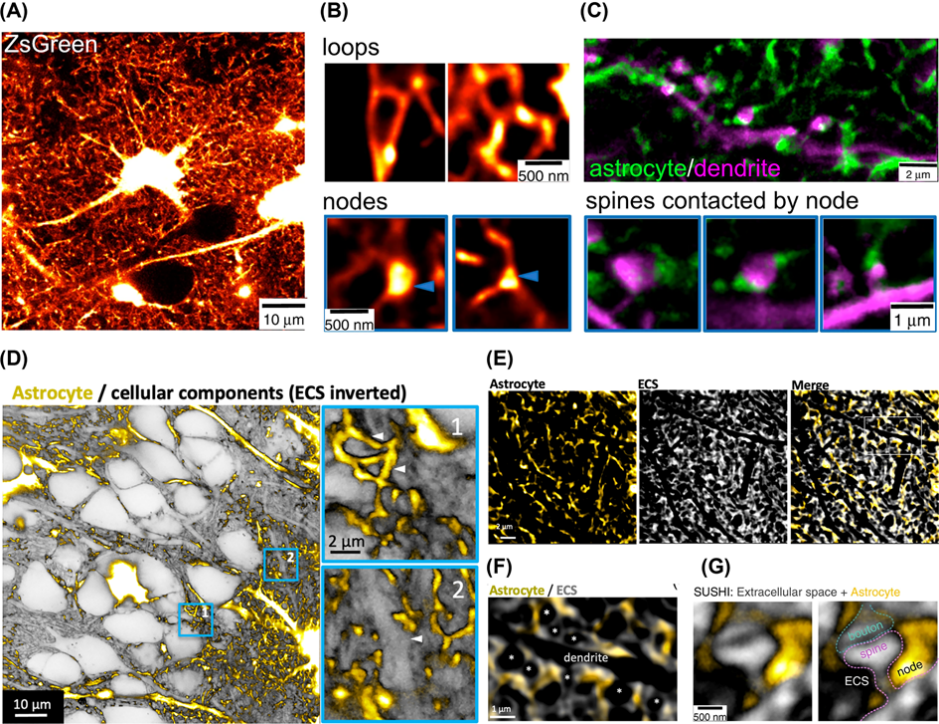
STED images depicting nanoscopic astrocyte structures in living organotypic slices (**A**) Confocal image of ZsGreen expressing astrocytes, highlighting the entire cell morphology; modified from [105] with permission. (**B**) STED images of astrocyte spongiform morphology, revealing specialised process structures – loops and nodes, modified from [105] with permission. (**C**) STED images of astrocyte (green) and dendrites (magenta), highlighting astrocytic nodes as the main contact points of spines; modified from [105] with permission. (**D**) STED image of astrocytes (gold) and inverted signal of extracellular space (ECS), highlighting astrocytic processes penetrating the neuropil; modified from [104] with permission. (**E**) STED images of positively labelled astrocyte (gold) and stained ECS (grey), modified from [104] with permission. (**F**) STED image of astrocyte (gold) and ECS (grey) depicting white rectangle in (E), highlighting a negative imprint of a putative dendrite and spines (*) (black), modified from [104] with permission. (**G**) STED image of tripartite synapse with astrocytic node in close apposition to spine, modified from [105] with permission.

As mentioned above, astroglia show diverse calcium signalling [21]. In a recent beautiful study, Nägerl and colleagues investigated the nanoscopic structure of PAPs and their localised Ca^2+^ transients using STED microscopy in living organotypic brain slices (Figure 3A–C) [105]. The researchers found that thin astrocytic processes are organised in a fine reticular network, exhibiting nodes, shafts and ring-like structures. The majority of Ca^2+^ transients takes place in nodes, and 55% of spines are in close apposition to at least one node. Node size as well as the size of the Ca^2+^ transients were positively correlated to spine size, highlighting the possibility that there are different astrocyte compartments handling different modes of signalling at node versus shaft tripartite synapses [105].

In a second study, Nägerl and colleagues used ‘super-resolution shadow imaging’ (SUSHI), a technique, in which the extracellular space is fluorescently labelled and cellular structures appear as dark shadows (Figure 3D–G) [104,105,122]. The researchers saw that the aforementioned ring-like structures that are formed by the reticular network of astrocytic processes, encircle spines and areas of interstitial fluid (Figure 3D–G) [104]. After osmotic challenge, the astrocyte structures swell, increasing the interface between astrocytic and other cellular structures but decreasing the size of interstitial pools [104].

## Concluding remarks

Super-resolution microscopy has helped further our understanding of neuronal and glial cell morphology and molecular dynamics. Most findings were acquired in fixed cell culture or brain section preparations. However, it is known that cultured cells differ from their *in vivo* counterparts. This is especially true for astroglia as their morphology and expression profiles are very different in culture and in intact tissue. Additionally, PAPs appear to be very sensitive to classical tissue fixation protocols, with a significant difference of PAP-positive synapses found in the adult murine neocortex when comparing chemical and cryo fixation (chemical 62% versus cryo: 34%) [123]. Adopting milder fixation methods and the development of improved *in vivo* imaging platforms will help evaluating the true morphology of astrocytes and hence tripartite synapses. We are only just beginning to fully explore the potential of super-resolution microscopy technology and its future capabilities, especially in the field of diagnostics.

As mentioned above, recent developments using STED microscopy have made *in vivo* nanoscopy possible. Further adaptations and the integration of adaptive optics [124] will improve image resolution and depth penetration, making imaging of highly dynamic and densely packed structures in living brain tissue more feasible. Implementations of adaptive optics have already shown promising results using in vivo two-photon excitation imaging [125–127], STED [128,129] and SMLM [130].

Astrocytes found in higher primates are larger and more complex, and show unique functions and different abilities to cope with stressors and disease responses when compared with murine astrocytes [131–134]. Super-resolution microscopy techniques will be essential in understanding the differences on a molecular level.

It is well documented that glial cells undergo drastic changes under pathological conditions [135]. Super-resolution imaging has already been applied for analysis of models of neurological and neurodegenerative diseases [136–139]. Additionally, it has also recently been utilised in human Alzheimer’s and Parkinson’s disease post-mortem tissue as a diagnostic tool [140,141].

Hence, the use of super-resolution will be a valuable means for both clinicians and researchers in deciphering the exact pathophysiological changes occurring in and around synapses, and in evaluating novel therapeutic options.

## Data Availability

No data were used for this review article.

## Abbreviations

AMPAR: α-amino-3-hydroxy-5-methyl-4-isoxazolepropionic acid receptors
BEST1: bestrophin-1
EM: electron microscopy
ExM: expansion microscopy
GABA: gamma-aminobutyric acid
NMDAR: N-methyl-D-aspartate receptors
NRCAM: neuronal cell adhesion molecule
PALM: photo-activated localisation microscopy
PAP: perisynaptic astrocytic process
PSD: postsynaptic density
SIM: structured illumination microscopy
SMLM: single-molecule localisation microscopy
SNARE: soluble N-ethylmaleimide-sensitive factor attachment protein receptor
sptPALM: single particle tracking PALM
STED: stimulated-emission depletion
STORM: stochastic optical reconstruction microscopy
SUSHI: super-resolution shadow imaging
uPAINT: universal point accumulation for imaging in nanoscale topography
